# Efficacy and safety of Ensitrelvir in asymptomatic or mild to moderate COVID-19: a systematic review and meta-analysis of randomized controlled trials

**DOI:** 10.1007/s15010-025-02582-0

**Published:** 2025-07-31

**Authors:** Muhammad Zain Ul Haq, Saad Ashraf, Muhammad Shahmeer Ullah Shah, Samia Aziz Sulaiman, Ayesha Shaukat, Muhammad Ahsan Ansari, Areeba Aamir Ali Basaria, Laveeza Fatima, Humza Saeed, Aman Goyal, Mohamed Daoud

**Affiliations:** 1 Department of Medicine, Dow University of Health Sciences, Karachi, Pakistan; 2 School of Medicine, University of Jordan, Amman, Jordan; 3 Allama Iqbal Medical College, Lahore, Pakistan; 4 Department of Internal Medicine, Rawalpindi Medical University, Rawalpindi, Pakistan; 5 Bogomolets National Medical University, Kyiv, Ukraine; 6 Department of Internal Medicine, Cleveland Clinic Foundation, Cleveland, USA

**Keywords:** Covid-19, Ensitrelvir, Pandemic, Meta-analysis

## Abstract

**Introduction:**

Since late 2019, COVID-19 has had a catastrophic impact on public health. Ensitrelvir, a new antiviral targeting the SARS-CoV-2 main protease, has reduced viral replication and disease severity. This meta-analysis and systematic review assessed Ensitrelvir’s efficacy and safety in patients with mild-to-moderate COVID-19 symptoms.

**Methods:**

A comprehensive search was conducted in PubMed (Medline), Scopus, Embase, and CENTRAL up to July 2024 to retrieve randomized controlled trials (RCTs) comparing Ensitrelvir to placebo in adults with mild to moderate, RT-PCR–confirmed COVID-19. Outcomes were assessed at standardized time points, with viral RNA measured at day 4. Mean differences (MD) for continuous outcomes and risk ratios (RR) for binary outcomes, both with 95% confidence intervals (CIs), were calculated using the Mantel–Haenszel random-effects model. Efficacy outcomes included SARS-CoV-2 viral RNA, while safety outcomes included HDL, triglycerides, bilirubin, AST, headache, diarrhea, TEAEs, TRAEs, serious TEAEs, and treatment discontinuation. The quality of the included RCTs was assessed with the Cochrane Risk of Bias 2 (ROB2) tool.

**Results:**

The analysis included six RCTs with 2,793 participants: 1,860 received Ensitrelvir and 933 were given a placebo. Ensitrelvir gave significant results for reduced viral RNA levels of SARS-CoV-2 [MD: − 1.35; 95% CI − 1.58 to − 1.13; p < 0.01] and the incidence of lower cholesterol levels [RR: 8.83; 95% CI 4.05 to 19.27; p < 0.01] compared to the placebo group. However, it was associated with increased risks of decreased HDL levels, elevated triglycerides, increased bilirubin, more headaches, and a higher overall occurrence of treatment-emergent adverse events.

**Conclusion:**

Ensitrelvir effectively reduces viral load in COVID-19 patients, but its safety profile raises concerns due to significant adverse effects. The benefits must be carefully weighed against the risks, and further research is needed to confirm its role in treatment and to find ways to mitigate these adverse effects.

**Supplementary Information:**

The online version contains supplementary material available at 10.1007/s15010-025-02582-0.

## Introduction

COVID-19 is a viral, infectious disease caused by the severe acute respiratory syndrome coronavirus 2 (SARS-CoV-2) [1]. SARS-CoV-2 primarily targets the respiratory system, yet may also affect other organs due to its systemic nature. The disease was first identified in Wuhan, China, in late 2019 and has caused a global pandemic, leading to lockdowns globally, dramatically affecting public health and economies worldwide [1]. As of mid-2024, COVID-19 has caused significant morbidity and mortality globally, with over 760 million confirmed cases and more than 6.9 million deaths [2]. This disease manifests with a broad range of symptoms, from mild respiratory issues to severe conditions such as pneumonia and acute respiratory distress syndrome (ARDS). The pathophysiology involves a hyperactive immune response, leading to elevated levels of inflammatory cytokines like IL-6, IL-1β, and TNF-α, contributing to the disease's severity [1]. Individuals older than 60 years and those with pre-existing conditions such as hypertension, diabetes, and heart disease are at higher risk for severe illness and mortality [3]. Apart from high-risk populations, the pandemic has also placed an immense strain on healthcare systems and the general population, with significant impacts on mental health, including increased anxiety, depression, and social isolation [4].

Current treatment strategies for mild to moderate COVID-19 primarily include supportive care, antiviral drugs, and immunomodulatory therapies. Remdesivir, an antiviral agent, has received emergency use authorization in several countries, while dexamethasone, a corticosteroid, has shown efficacy in reducing mortality in severe cases [5, 6]. Nirmatrelvir/ritonavir (Paxlovid), an oral antiviral combination, is also approved for mild-to-moderate COVID-19 in high-risk patients, demonstrating significant reductions in hospitalization and mortality, particularly during the Delta variant phase. However, Paxlovid requires co-administration of ritonavir, which may lead to drug-drug interactions, complicating its use in patients with comorbidities or polypharmacy [7]*.* Moreover, these treatments have limitations and may be associated with side effects such as gastrointestinal issues and immune suppression, which could lead to complications [8]. The search for additional practical and safe therapies continues, especially for patients with less severe forms of the disease who may benefit from early and targeted interventions.

Ensitrelvir, a novel antiviral agent, targets the replication of SARS-CoV-2 by inhibiting the main protease (Mpro) enzyme, which is essential for viral replication. This mechanism reduces viral load and alleviates disease severity [9, 10]. Though these preliminary trials show potential benefits of using Ensitrelvir, concerns remain over its efficacy and safety. Previous randomized clinical trials have been limited by smaller sample sizes and shorter follow-up periods, which may lead to inconsistencies in results. Therefore, our meta-analysis and systematic review aim to address this gap by systematically reviewing and analyzing available clinical trial data on Ensitrelvir, thoroughly assessing its efficacy and safety.

## Methods

This meta-analysis and systematic review was conducted in accordance with the Preferred Reporting Items for Systematic Reviews and Meta-Analyses (PRISMA) guidelines [11]. The protocol for this meta-analysis was registered in the International Prospective Register of Systematic Reviews (PROSPERO) under the reference number CRD42024572306.

### Data sources and search strategy

A comprehensive literature search was performed across Cochrane CENTRAL, PubMed/MEDLINE, and Google Scholar databases from inception to July 2024. The search strategy imposed no restrictions on publication status or language. Additionally, bibliographies of relevant review articles and databases of unpublished literature were searched to ensure that no relevant studies, including those from white or gray literature, were omitted. The search terms included relevant PubMed MeSH terms and related keywords, such as (Ensitrelvir OR S-217622 OR 3CL protease inhibitor) AND (COVID-19 OR SARS-CoV-2 OR). The detailed search strategy is provided in Supplementary Table 1.

### Study selection and eligibility criteria

Retrieved studies were imported into EndNote reference library, version X8.1 (Clarivate Analytics), and duplicates were removed. Two authors (ZH and SS) independently reviewed and selected studies. Discrepancies were resolved by a third author (SA). The Population, Intervention, Control, and Outcomes (PICO) format for systematic reviews was used to define the inclusion criteria, with 'P' representing patients with COVID-19 positivity, 'I' representing Ensitrelvir, 'C' representing placebo, and 'O' representing the several outcomes defined below. Studies that met all of the following criteria were included in the meta-analysis and systematic review: (1) Randomized controlled trials (RCTs), (2) comparing Ensitrelvir to placebo, (3) in a population of patients with mild to moderate COVID-19 symptoms, and (4) reporting any of the prespecified outcomes of interest. Only studies explicitly identified as randomized controlled trials in study design descriptions were included. These RCTs compared Ensitrelvir to placebo in adults (≥ 18 years) with mild-to-moderate, RT-PCR-confirmed COVID-19.

We excluded preprints and included only peer-reviewed, published randomized controlled trials. Studies were excluded if they employed different study designs, such as observational studies, non-randomized studies, review articles, case reports, or editorials. Observational studies were excluded to minimize confounding and bias inherent in non-randomized designs, ensuring higher internal validity of the findings through RCTs. Additionally, studies with overlapping populations, those not reporting relevant outcomes, and those involving patients with exacerbated COVID-19 or other serious systemic comorbidities were also excluded.

Serious systemic comorbidities were defined as conditions increasing COVID-19 severity risk, such as uncontrolled diabetes, severe cardiovascular disease, chronic kidney disease, or immunosuppression. Studies with populations predominantly having these conditions were excluded to ensure homogeneity and maintain a focus on mild-to-moderate COVID-19.Key endpoints include a decrease in SARS-CoV-2 viral RNA on day four. Safety endpoints include liver enzymes (bilirubin, AST), lipid abnormalities (HDL, triglycerides), and adverse events (TEAEs, TRAEs) that were monitored during treatment.

### Data extraction

Data extraction was conducted independently by two authors (ZH and SS) on a pre-piloted Microsoft Excel sheet based on a subset of studies to refine the extraction form before complete data collection, with discrepancies resolved through consultation with a third author (SA). Extracted data included study labels, publication year, study design, location, patient population, baseline characteristics (age, sex, disease duration), and efficacy and safety endpoints. The efficacy endpoints evaluated the impact on SARS-CoV-2 viral RNA levels, with safety endpoints including various adverse events (AEs), including a decrease in blood high-density lipoproteins (HDL) and cholesterol levels, an increase in blood triglyceride levels, elevated bilirubin and aspartate aminotransferase (AST) levels, headache, diarrhea, treatment-emergent adverse events (TEAEs), treatment-related adverse events (TRAEs), serious TEAEs, and AEs leading to treatment discontinuation.

### Quality assessment

The quality of the included RCTs was assessed using the Cochrane risk of bias tool 2.0 (RoB 2.0) [12]. The tool evaluates potential biases in domains such as selection, performance, detection, attrition, and reporting, with judgments categorized as 'Low', 'Some concerns' or 'High'. Two authors (SS and SA) independently performed the quality assessment, resolving disagreements through consensus. The certainty of evidence for primary efficacy (SARS-CoV-2 viral RNA levels) and safety outcomes (e.g., HDL decrease, TEAEs) was evaluated using the Grading of Recommendations Assessment, Development, and Evaluation (GRADE) approach. Two authors (SA, ZH) independently assessed the risk of bias, inconsistency, indirectness, imprecision, and publication bias (Supplementary Table 2).

### Statistical analysis

Statistical analysis was performed using Review Manager (RevMan version 5.4; Copenhagen: The Nordic Cochrane Centre, The Cochrane Collaboration, 2014). The Mantel–Haenszel random-effects model was used to pool the outcomes from the studies. Mean difference (MD) for continuous outcomes and Risk Ratio (RR) for binary outcomes, along with their 95% confidence intervals (CIs), were calculated. Statistical significance was defined as a p value ≤ 0.05. Statistical heterogeneity was assessed using the Higgins I^2^ index, with values < 25%, 25–75%, and > 75% representing low, moderate, and high heterogeneity, respectively. In instances where heterogeneity exceeded 25%, a leave-one-out sensitivity analysis was performed using a leave-one-out approach to identify the study contributing to significant heterogeneity.

## Results

### Study selection

**Fig. 1:**
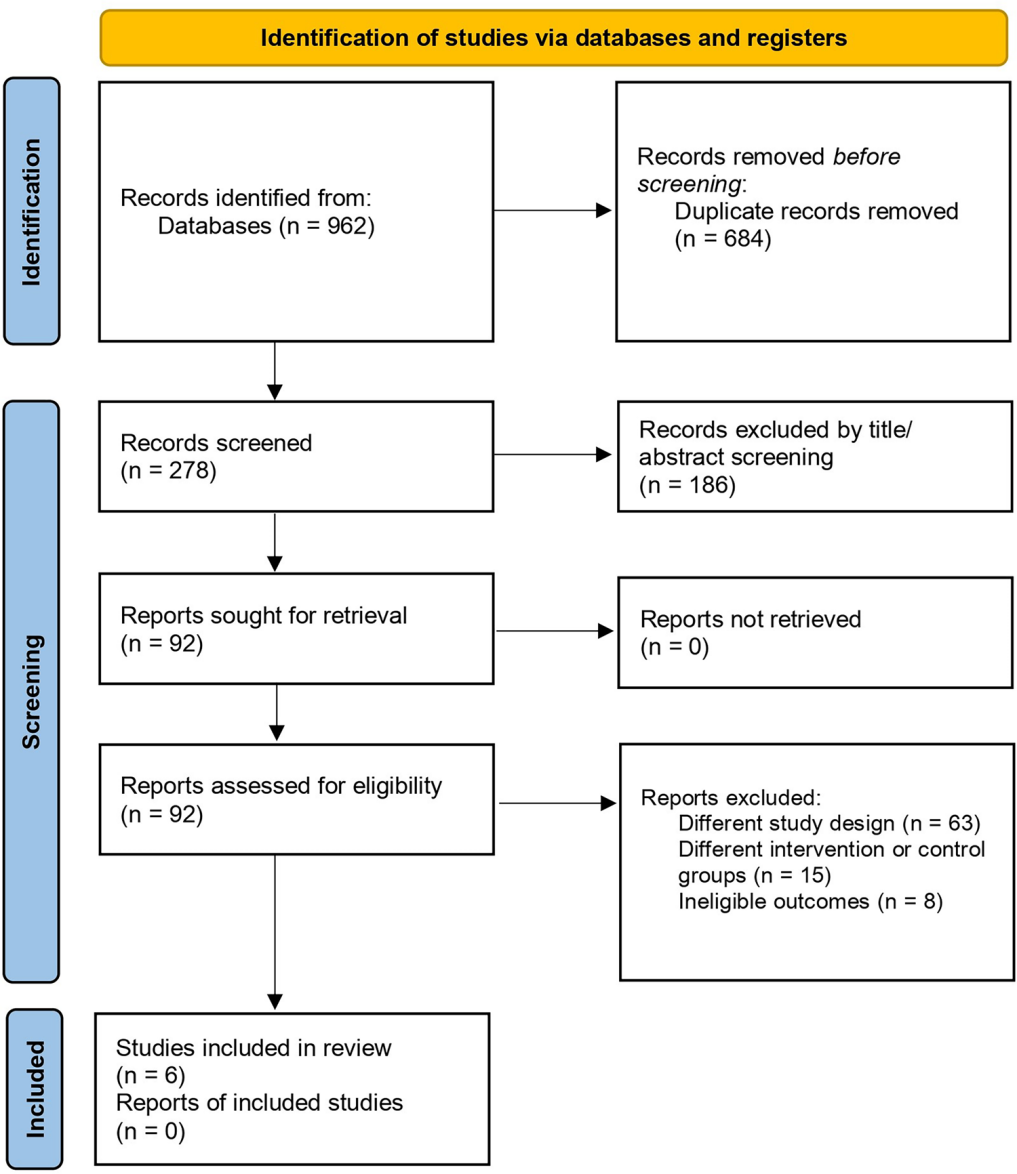
The 2020 preferred reporting items for systematic reviews and meta-analyses (PRISMA) flowchart

The preliminary literature search from databases yielded 962 results. After removing duplicates (n = 684), an initial screening was performed based on the titles and abstracts of the remaining articles, narrowing the selection to 92 articles. Further screening led to the exclusion of 63 articles due to different study designs, 15 due to different intervention or control groups, and 8 due to ineligible outcomes. Ultimately, 6 trials met the predefined inclusion criteria and were included in this meta-analysis [10, 13–17]. The search and screening process is illustrated in the PRISMA flow chart (Fig. 1)**.**

### Study and patient characteristics

A total of 2,793 patients were included in the six RCTs, with 1,860 in the Ensitrelvir group and 933 in the placebo group. Among these participants, 1,510 were male, and 1,283 were female. The studies included 24 participants identified as White and 2,362 as Asian. The mean age of the patients was 37.0 ± 11.5 years. Across the included studies, the mean proportion of male participants was approximately 55.8%, with values ranging from 50.0 to 76.5%, indicating a slight male predominance in the study populations. The studies included the assessment of the Delta variant, the Omicron BA.1 lineage (21K), the Omicron BA.2 lineage (211), and unidentified, unknown, or recombinant variants. Efficacy outcomes, including SARS-CoV-2 viral RNA levels, were assessed at day 4 post-treatment, while safety outcomes, such as treatment-emergent adverse events (TEAEs), lipid abnormalities (e.g., HDL, triglycerides), and liver enzyme elevations (e.g., bilirubin, AST), were monitored up to day 28 across all included studies. The baseline characteristics and outcomes of the studies included are detailed in Tables 1, 2 and 3**,** respectively.

**Table 1:** Baseline characteristics

Baseline characteristics	Author names								
	Mukae et al. 2023			Shimizu et al. 2023			Ohmagari et al. 2024		
	125 mg	250 mg	Placebo	125 mg	250 mg	Placebo	125 mg	250 mg	Placebo
Total population	114	111	116	8	8	8	194	189	189
Male sex, n (%)	61 (53.5)	66 (56.9)	72 (64.9)	4 (50.0)	4 (50.0)	4 (50.0)	109 (56.2)	110 (58.2)	103 (54.5)
Age, mean (SD), years	35.6 (13.5)	35.3 (13.1)	37.3 (12.6)	33.8 (3.3)	38.1 (8.1)	33.5 (7.1)	37.9 (12.0)	40.9 (13.4)	38.6 (13.0)
Height (cm) mean (SD)	N/A	N/A	N/A	167.6 (11.2)	171.8 (7.7)	169.9 (9.9)	N/A	N/A	N/A
Weight (kg) mean (SD)	N/A	N/A	N/A	70.6 (11.1)	73.5 (12.4)	68.4 (6.2)	N/A	N/A	N/A
BMI (kg/m^2^) mean (SD)	N/A	N/A	N/A	24.7 (1.3)	24.7 (2.6)	23.9 (2.9)	N/A	N/A	N/A
Race, n (%)	N/A	N/A	N/A	White: 8 (100.0)	White: 8 (100.0)	White: 8 (100.0)	Asian:193 (99.5)	Asian:189 (100.0)	Asian:188 (99.5)
COVID-19 vaccination history, n (%):									
≥ 1 vaccination	97 (85.1)	97 (83.6)	97 (87.4)	N/A	N/A	N/A	N/A	N/A	N/A
≥ 2 vaccination	94 (82.5)	96 (82.8)	95 (85.6)	N/A	N/A	N/A	N/A	N/A	N/A
≥ 3 vaccination	3 (2.6)	1 (0.9)	2 (1.8)	N/A	N/A	N/A	N/A	N/A	N/A
Overall	N/A	N/A	N/A	N/A	N/A	N/A	178 (91.8)	173 (91.5)	174 (92.1)
SARS-CoV-2 variant, n (%):									
21I (Delta)	0 (0.0)	1 (0.9)	0 (0.0)	N/A	N/A	N/A	1 (0.5)	0 (0.0)	0 (0.0)
21J (Delta)	0 (0.0)	1 (0.9)	0 (0.0)	N/A	N/A	N/A	0 (0.0)	0 (0.0)	1 (0.5)
21K (Omicron BA.1 lineage)	114 (100.0)	112 (96.6)	110 (99.1)	N/A	N/A	N/A	53 (27.3)	47 (24.9)	50 (26.5)
21L (Omicron BA.2 lineage)	0 (0.0)	1 (0.9)	1 (0.9)	N/A	N/A	N/A	71 (36.6)	68 (36.0)	53 (28.0)
Unknown/unidentified/recombinant	0 (0.0)	1 (0.9)	0 (0.0)	N/A	N/A	N/A	53 (27.3)	58 (30.7)	63 (33.3)
Respiratory symptoms									
Stuffy or runny nose	29 (25.4)	34 (29.3)	26 (23.4)	N/A	N/A	N/A	N/A	N/A	N/A
Sore throat	65 (57.0)	63 (54.3)	54 (48.6)	N/A	N/A	N/A	N/A	N/A	N/A
Shortness of breath (difficulty breathing)	8 (7.0)	8 (6.9)	1 (0.9)	N/A	N/A	N/A	N/A	N/A	N/A
Cough	48 (42.1)	46 (39.7)	49 (44.1)	N/A	N/A	N/A	N/A	N/A	N/A
Total score of 12 COVID-19 symptoms, b mean (SD)	9.9 (5.0)	9.3 (4.5)	8.6 (3.8)	N/A	N/A	N/A	N/A	N/A	N/A
Patients with fever (body temperature ≥ 37.0 °C), n (%)	45 (39.5)	39 (33.6)	30 (27.0)	N/A	N/A	N/A	N/A	N/A	N/A
SARSCoV-2 test, negative	N/A	N/A	N/A	8 (100.0)	8 (100.0)	8 (100.0)	N/A	N/A	N/A
SARS-CoV-2 viral RNA level (log10 copies/mL), mean (SD)	N/A	N/A	N/A	N/A	N/A	N/A	6.43 (1.26)	6.56 (1.15)	6.18 (1.48)
Prior acetaminophen use	N/A	N/A	N/A	N/A	N/A	N/A	45 (23.2)	56 (29.6)	44 (23.3)
Any risk factor for severe disease	N/A	N/A	N/A	N/A	N/A	N/A	58 (29.9)	63 (33.3)	66 (34.9)

Baseline characteristics	Author names								
	Yotsuyanag et al. 2022			Mukae et al. 2022			Shimizu et al. 2022		
	125 mg	250 mg	Placebo	125 mg	250 mg	Placebo	125 mg	250 mg	Placebo
Total population	603	595	600	16	14	17	8	–	3
Male sex, n (%)	318 (52.7)	323 (54.3)	311 (51.8)	8 (50.0)	8 (57.1)	13 (76.5)	N/A	–	N/A
Age, mean (SD), years	35.9 (12.7)	35.9 (12.7)	35.3 (12.6)	38.8 (12.5)	40.4 (10.7)	38.0 (14.2)	N/A	–	N/A
Height (cm) mean (SD)	N/A	N/A	N/A	N/A	N/A	N/A	181.01 (9.58)	–	181.63 (7.28)
Weight (kg) mean (SD)	N/A	N/A	N/A	N/A	N/A	N/A	74.68 (8.32)	–	81.13 (3.23)
BMI (kg/m^2^) mean (SD)	N/A	N/A	N/A	N/A	N/A	N/A	22.83 (2.91)	–	24.40 (2.00)
Race, n (%)	Asian: 601 (99.7)	Asian: 593 (99.7)	Asian: 598 (99.7)	N/A	N/A	N/A	8 (100.0)	–	3 (100.0)
COVID-19 vaccination history, n (%):									
≥ 1 vaccination	562 (93.2)	551 (92.6)	553 (92.2)	N/A	N/A	N/A	N/A	–	N/A
≥ 2 vaccination	554 (91.9)	547 (91.9)	545 (90.8)	N/A	N/A	N/A	N/A	–	N/A
≥ 3 vaccination	284 (47.1)	295 (49.6)	283 (47.2)	N/A	N/A	N/A	N/A	–	N/A
Overall	N/A	N/A	N/A	14 (87.5)	12 (85.7)	12 (70.6)	N/A	–	N/A
SARS-CoV-2 variant, n (%):									
21I (Delta)	N/A	N/A	N/A	Delta (subvariant: N/A): 13 (81.3)	Delta (subvariant: N/A): 13 (92.9)	Delta (subvariant: N/A): 16 (94.1)	N/A	–	N/A
21J (Delta)	N/A	N/A	N/A	N/A	N/A	N/A	N/A	–	N/A
21K (Omicron BA.1 lineage)	131 (21.7)	130 (21.8)	115 (19.2)	Omicron (subvariant: N/A): 3 (18.8)	Omicron (subvariant: N/A): 1 (7.1)	Omicron (subvariant: N/A): 1 (5.9)	N/A	–	N/A
21L (Omicron BA.2 lineage)	401 (66.5)	376 (63.2)	407 (67.8)	N/A	N/A	N/A	N/A	–	N/A
Unknown/unidentified/recombinant	52 (8.6)	64 (10.8)	50 (8.3)	N/A	N/A	N/A	N/A	–	N/A
Respiratory symptoms									
Stuffy or runny nose	506 (86.1)	468 (80.6)	464 (80.0)	6 (46.2)	4 (33.3)	4 (28.6)	N/A	–	N/A
Sore throat	520 (88.4)	522 (89.7)	512 (88.3)	7 (53.8)	2 (16.7)	3 (21.4)	N/A	–	N/A
Shortness of breath (difficulty breathing)	170 (28.9)	158 (27.1)	142 (24.5)	1 (7.7)	0 (0.0)	0 (0.0)	N/A		N/A
Cough	518 (88.1)	512 (88.0)	518 (89.3)	7 (53.8)	7 (58.3)	6 (42.9)	N/A		N/A
Total score of 12 COVID-19 symptoms, b mean (SD)	N/A	N/A	N/A	N/A	N/A	N/A	N/A		N/A
Patients with fever (body temperature ≥ 37.0 °C), n (%)	N/A	N/A	N/A	N/A	N/A	N/A	N/A		N/A
SARSCoV-2 test, negative	N/A	N/A	N/A	N/A	N/A	N/A	8 (100.0)		3 (100.0)
SARS-CoV-2 viral RNA level (log10 copies/mL), mean (SD)	6.83 (1.05)	6.73 (1.08)	6.77 (1.07)	N/A	N/A	N/A	N/A		N/A
Prior acetaminophen use	216 (35.8)	191 (32.1)	N/A	N/A	N/A	N/A	N/A		N/A
Any risk factor for severe disease	174 (28.9)	167 (28.1)	152 (25.3)	N/A	N/A	N/A	N/A		N/A

**Table 2:** Safety outcomes

Safety outcomes	Author names																	
	Mukae et al. 2023			Shimizu et al. 2023			Ohmagari et al. 2024			Yotsuyanag et al. 2022			Mukae et al. 2022			Shimizu et al. 2022		
	125 mg	250 mg	Placebo	125 mg	250 mg	Placebo	125 mg	250 mg	Placebo	125 mg	250 mg	Placebo	125 mg	250 mg	Placebo	125 mg	250 mg	Placebo
Patients with any TEAE	48/140 (34.3%)	60/140 (42.9%)	44/141 (31.2%)	7/8 (87.5%)	7/8 (87.5%)	3/8 (37.5%)	88/201 (43.8%)	115/202 (56.9%)	43/201 (21.4%)	267/604 (44.2%)	321/599 (53.6%)	150/605 (24.8%)	11/21 (52.4%)	16/23 (69.6%)	9/24 (37.5%)	–	8/8 (100%)	1/3 (33.3%)
Patients with any serious TEAE	0 (0.0%)	0 (0.0%)	2 (1.4%)	N/A	N/A	N/A	0 (0.0%)	2 (1.0%)	0 (0.0%)	1 (0.2%)	0 (0.0%)	1 (0.2%)	0 (0.0%)	0 (0.0%)	0 (0.0%)	–	N/A	N/A
Patients with any treatment-related AE	19 (13.6%)	31 (22.1%)	7 (5.0%)	N/A	N/A	N/A	47 (23.4)	75 (37.1)	14 (7.0)	148 (24.5%)	217 (36.2%)	60 (9.9%)	5 (23.8%)	10 (43.5%)	0 (0.0%)	–	N/A	N/A
Patients with TEAEs leading to death	0 (0.0%)	0 (0.0%)	0 (0.0%)	N/A	N/A	N/A	0 (0.0%)	0 (0.0%)	0 (0.0%)	0 (0.0%)	0 (0.0%)	0 (0.0%)	0 (0.0%)	0 (0.0%)	0 (0.0%)	–	N/A	N/A
Patients with TEAEs leading to treatment discontinuation	2 (1.4%)	0 (0.0%)	0 (0.0%)	N/A	N/A	N/A	1 (0.5%)	2 (1.0%)	0 (0.0%)	4 (0.7%)	6 (1.0%)	2 (3.0%)	0 (0.0%)	0 (0.0%)	0 (0.0%)	–	N/A	N/A
Blood triglycerides increase	1 (0.7%)	9 (6.4%)	1 (0.7%)	0/8 (0.0%)	1/8 (12.5%)	0/8 (0.0%)	14 (7.0%)	22 (10.9%)	9 (4.5%)	49 (8.1%)	74 (12.4%)	32 (5.3%)	0 (0.0%)	3 (13.0%)	0 (0.0%)	–	0 (0.0%)	0 (0.0%)
HDL decrease	31 (22.1%)	40 (28.6%)	5 (3.5%)	5 (62.5%)	7 (87.5%)	0 (0.0%)	61 (30.3%)	91 (45.0%)	4 (2.0%)	188 (31.1%)	231 (38.6%)	23 (3.8%)	3 (14.3%)	12 (52.2%)	2 (8.3%)	–	7/8 (87.5%)	0 (0.0%)
Blood creatine phosphokinase increase	0 (0.0%)	0 (0.0%)	4 (2.8%)	0 (0.0%)	0 (0.0%)	0 (0.0%)	N/A	N/A	N/A	14 (2.3)	8 (1.3%)	11 (1.8%)	N/A	N/A	N/A	–	0 (0.0%)	0 (0.0%)
Diarrhea	2 (1.4%)	3 (2.1%)	1 (0.7%)	2 (25.0%)	1 (12.5%)	0 (0.0%)	1 (0.5%)	6 (3.0%)	4 (2.0%)	6 (1.0%)	9 (1.5%)	12 (2.0%)	N/A	N/A	N/A	–	4/8 (50%)	1/3 (33.3%)
Rash	2 (1.4%)	1 (0.7%)	3 (2.1%)	N/A	N/A	N/A	N/A	N/A	N/A	N/A	N/A	N/A	N/A	N/A	N/A	–	0 (0.0%)	0 (0.0%)
Back pain	1 (0.7%)	3 (2.1%)	1 (0.7%)	N/A	N/A	N/A	N/A	N/A	N/A	N/A	N/A	N/A	N/A	N/A	N/A	–	N/A	N/A
Headache	3 (2.1%)	3 (2.1%)	0 (0.0%)	0 (0.0%)	3 (37.5%)	2 (25.0%)	5 (2.5%)	11 (5.4%)	3 (1.5%)	13 (2.2%)	20 (3.3%)	14 (2.3%)	1 (4.8)	3 (13.0)	0 (0.0%)	–	2/8 (25%)	0 (0.0%)
Nausea	N/A	N/A	N/A	0 (0.0%)	0 (0.0%)	0 (0.0%)	1 (0.5%)	7 (3.5%)	0 (0.0%)	N/A	N/A	N/A	N/A	N/A	N/A	–	0 (0.0%)	0 (0.0%)
Blood bilirubin increased	N/A	N/A	N/A	N/A	N/A	N/A	7 (3.5%)	15 (7.4%)	0 (0.0%)	36 (6.0%)	56 (9.3%)	6 (1.0%)	0 (0.0%)	2 (8.7%)	0 (0.0%)	–	N/A	N/A
Alanine Aminotransferase Increased	N/A	N/A	N/A	N/A	N/A	N/A	N/A	N/A	N/A	N/A	N/A	N/A	1 (4.8%)	0 (0.0%)	2 (8.3%)	–	N/A	N/A
Aspartate Aminotransferase Increased	N/A	N/A	N/A	N/A	N/A	N/A	N/A	N/A	N/A	4 (0.7%)	9 (1.5%)	12 (2.0%)	4 (0.7)	9 (1.5%)	12 (2.0%)	–	N/A	N/A
Blood cholesterol decreased	N/A	N/A	N/A	N/A	N/A	N/A	8 (4.0%)	7 (3.5%)	0 (0.0%)	20 (3.3%)	28 (4.7%)	3 (0.5%)	20 (3.3%)	28 (4.7%)	3 (0.5%)	–	N/A	N/A

**Table 3:** Efficacy outcomes

Author names	Year	Efficacy outcomes																							
		Time-weighted average change from baseline up to 120 hours in the total score of 12 COVID-19 symptoms									SARS-CoV-2 viral RNA level (day4)						Time to first improvement in COVID-19 symptoms			Time to first negative SARS-CoV-2 viral titer					
		Mean (SD) change from baseline			LS mean (SE) change from baseline assessed by ANCOVA			LS mean (SE) difference in change from baseline versus placebo			LS mean (SE) change from baseline assessed by ANCOVA			Difference from placebo			Unit: (median [95% CI] hours)			Median (h)			Difference from placebo		
		125 mg	250 mg	Placebo	125 mg	250 mg	Placebo	125 mg	250 mg	Placebo	125 mg	250 mg	Placebo	125 mg	250 mg	Placebo	125 mg	250 mg	Placebo	125 mg	250 mg	Placebo	125 mg	250 mg	Placebo
Mukae et al.	2023	− 5.95 (47.56)	− 5.42 (43.77)	− 4.92 (38.59)	− 5.37 (0.24)	− 5.17 (0.23)	− 5.12 (0.24)	− 0.24 (0.30), (95% CI, − 0.83 to 0.34); p-value: 0.4171	− 0.04 (0.29) (95% CI, − 0.62 to 0.53); p-value: 0.8806	Referent	− 2.58 [1.30]	− 2.49 [1.30]	− 1.28 [1.30]	− 1.30; 95% CI: − 1.57 to − 1.03; p < 0.0001	− 1.21; (95% CI, − 1.48 to − 0.94); p < 0.0001	Referent	28.0 [21.5–36.6]	27.8 [24.6–40.0]	36.6 [28.0–40.8]	47.7 [95%CI 43.4, 61.0]	47.4 [95%CI 42.8, 65.4]	91.6 [95%CI 74.1, 109.7]	− 43.8 [− 57.2, − 26.9] p = < 0.0001	− 44.1 [− 52.1, − 21.0] p = < 0.0001	Referent
Shimizu et al.	2023	N/A	N/A	N/A	N/A	N/A	N/A	N/A	N/A	N/A	N/A	N/A	N/A	N/A	N/A	N/A	N/A	N/A	N/A	N/A	N/A	N/A	N/A	N/A	N/A
Ohmagari et al.	2024	N/A	N/A	N/A	N/A	N/A	N/A	N/A	N/A	N/A	N/A	N/A	N/A	N/A	N/A	N/A	N/A	N/A	N/A	38.3 (95%CI 21.8, 42.3)	38.9 (95%CI 24.8, 43.2)	66.7 (95%CI 62.0, 71.2)	− 28.3 (95%CI − 47.5, − 22.0) p = < 0.0001	− 27.8 (95%CI − 45.5, − 21.6) p = < 0.0001	Referent
Yotsuyanag et al.	2022	N/A	N/A	N/A	N/A	N/A	N/A	N/A	N/A	N/A	− 2.48 (1.96)	− 2.49 (1.95)	− 1.01 (1.96)	− 1.47 (1.96) [95% CI: − 1.63 to − 1.31] p = < 0.001	− 1.48 (1.96) [95%CI − ; 1.64 to − 1.32] p = < 0.001	Referent	N/A	N/A	N/A	Median: 36.2 h; (95% CI, 23.4–43.2)	N/A	Median, 65.3 h; (95% CI, 62.0–66.8)	− 29.1 h; (95% CI, − 42.3 to − 21.1; p < 0.001)	N/A	Referent
Mukae et al.	2022	N/A	N/A	N/A	N/A	N/A	N/A	N/A	N/A	N/A	Mean (SD): − 2.42 [1.42]	Mean (SD): − 2.81 [1.21]	Mean (SD): − 1.54 [0.74]	− 0.88; (p = 0.0712)	− 1.27; (p = 0.0083)	Referent	N/A	N/A	N/A	61.3 (38.0, 68.4)	62.7 (39.2, 72.3)	111.1 (23.2, 158.5)	− 48.4 (− 95.9, 28.5) p = 0.0205	− 48.8 (− 96.7, 30.9) p = 0.0159	Referent
Shimizu et al.	2022	N/A	N/A	N/A	N/A	N/A	N/A	N/A	N/A	N/A	N/A	N/A	N/A	N/A	N/A	N/A	N/A	N/A	N/A	N/A	N/A	N/A	N/A	N/A	N/A

### Assessment of study quality

Four randomized controlled trials (RCTs) were classified as “low risk” [10, 13, 14, 17], while two were identified as having "some concerns" due to concerns in the missing outcome data, measurement of outcomes and selection of reported results (Supplementary Figs. 1 and 2) [15, 16]

### Endpoints

#### SARS-CoV-2 viral RNA levels

**Fig. 2:**
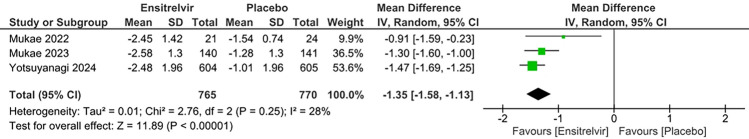
Forest plot of the change in SARS-CoV-2 viral RNA levels in the Ensitrelvir and placebo groups

Three studies reported the change in SARS-CoV-2 viral RNA levels in the Ensitrelvir and placebo groups [10, 16, 17]. According to the pooled analysis shown in Fig. 2, there was a significant reduction in viral RNA levels favoring Ensitrelvir over placebo [MD: − 1.35; 95% CI − 1.58 to − 1.13; p < 0.01; I^2^ = 28%]. There was moderate heterogeneity among the studies, which was reduced to zero after leaving out Mukae 2022 [16]

#### Decrease in blood cholesterol levels

**Fig. 3:**
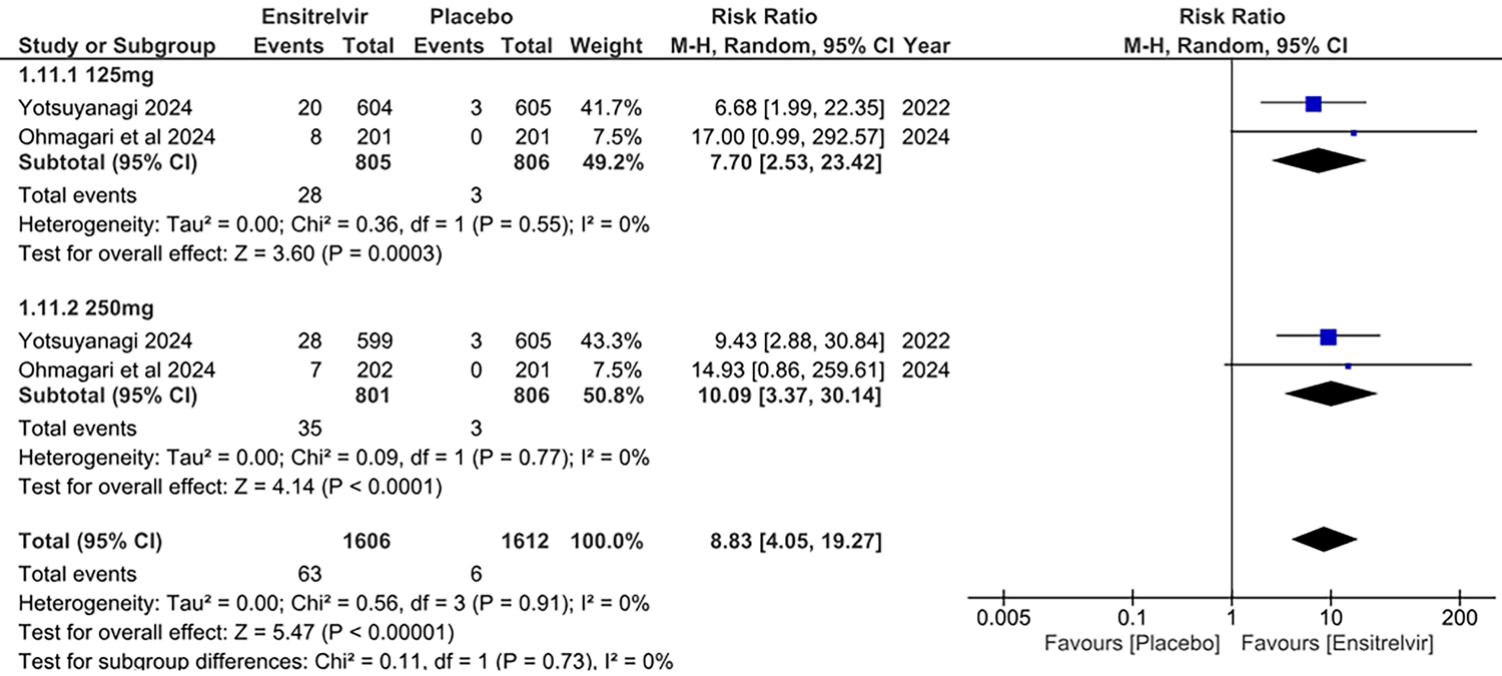
Forest plot of the decreased blood cholesterol levels in the Ensitrelvir and placebo groups

Two studies reported a decrease in blood cholesterol levels as an outcome [10, 13]. Figure 3 illustrates a pooled analysis which showed significant results in the Ensitrelvir group for the decrease in blood cholesterol levels with the 125 mg dose [RR: 7.70; 95% CI 2.53 to 23.42; p < 0.01; I^2^ = 0%] and 250 mg dose [RR: 10.09; 95% CI 3.37 to 30.14; p < 0.01; I^2^ = 0%] compared to placebo. The overall analysis similarly gave significant results in the Ensitrelvir group compared to placebo [RR: 8.83; 95% CI 4.05 to 19.27; p < 0.01; I^2^ = 0%]. There was no heterogeneity among the studies, showing highly consistent results.

#### Decrease in blood high-density lipoprotein (HDL) levels

**Fig. 4:**
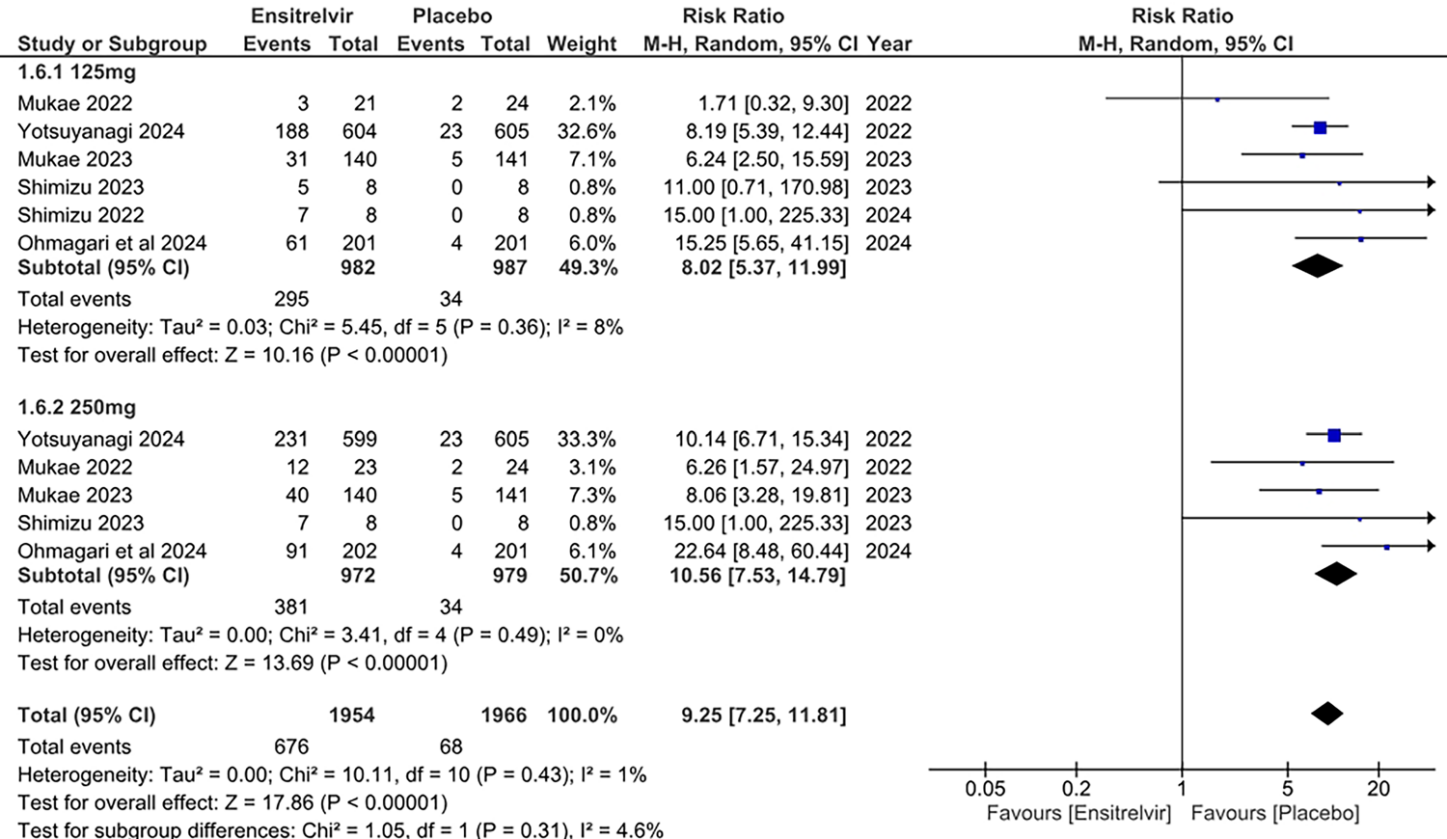
Forest plot of the decreased blood HDL levels in the Ensitrelvir and Placebo groups

Six studies examined the impact of Ensitrelvir at doses of 125 and 250 mg on blood HDL levels, comparing it to placebo [10, 13–17]. The pooled analysis in Fig. 4 demonstrated significantly higher risk for decrease in HDL levels with the 125 mg dose of Ensitrelvir [RR: 8.02; 95% CI 5.37 to 11.99; p < 0.01; I^2^ = 8%] and 250 mg dose [RR: 10.56; 95% CI 7.53 to 14.79; p < 0.01; I^2^ = 0%]. The combined analysis also followed the same trend, showing higher risk for reduction in HDL levels with Ensitrelvir treatment compared to placebo [RR: 9.25; 95% CI 7.25 to 11.81; p < 0.01; I^2^ = 1%]. The studies showed an overall low heterogeneity (I^2^) of 1%, reflecting very consistent results.

#### Increase in blood triglyceride levels

**Fig. 5:**
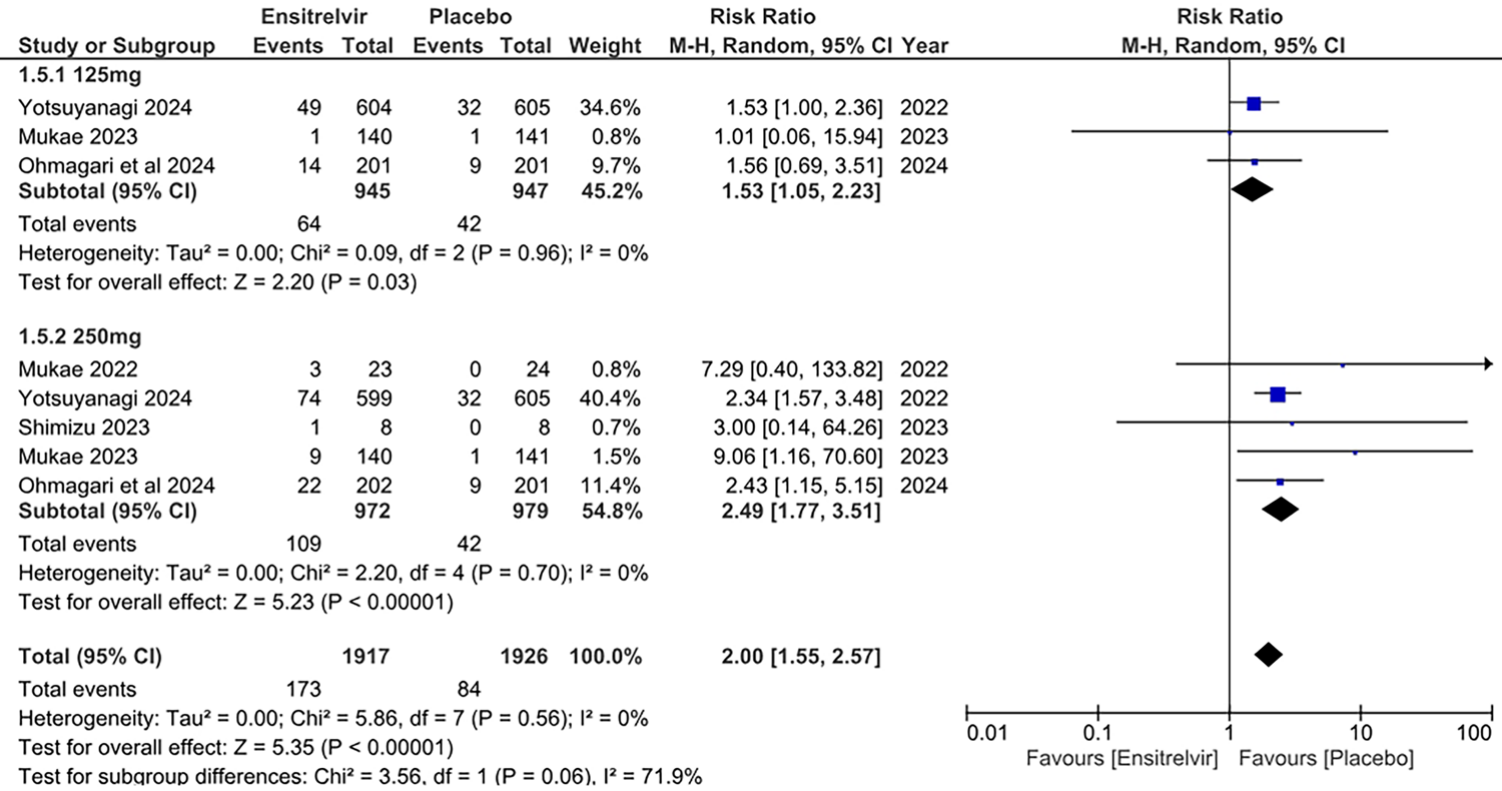
Forest plot of the increased blood triglyceride levels in the Ensitrelvir and placebo groups

Five studies reported the effects of Ensitrelvir at two doses (125 and 250 mg) on blood triglyceride levels [10, 13, 14, 16, 17]. The pooled analysis presented in Fig. 5 showed significantly higher risk of increase in blood triglyceride levels for both 125 mg dose of Ensitrelvir [RR: 1.53; 95% CI 1.05 to 2.23; p = 0.03; I^2^ = 0%] and 250 mg dose [RR: 2.49; 95% CI 1.77 to 3.51; p < 0.01; I^2^ = 0%] as compared to placebo. The combined analysis indicated a similar pattern with a higher risk of increased blood triglyceride levels with Ensitrelvir compared to placebo [RR: 2.00; 95% CI 1.55 to 2.57; p < 0.01; I^2^ = 0%]. No heterogeneity was observed among the studies.

#### Increase in bilirubin levels

**Fig. 6:**
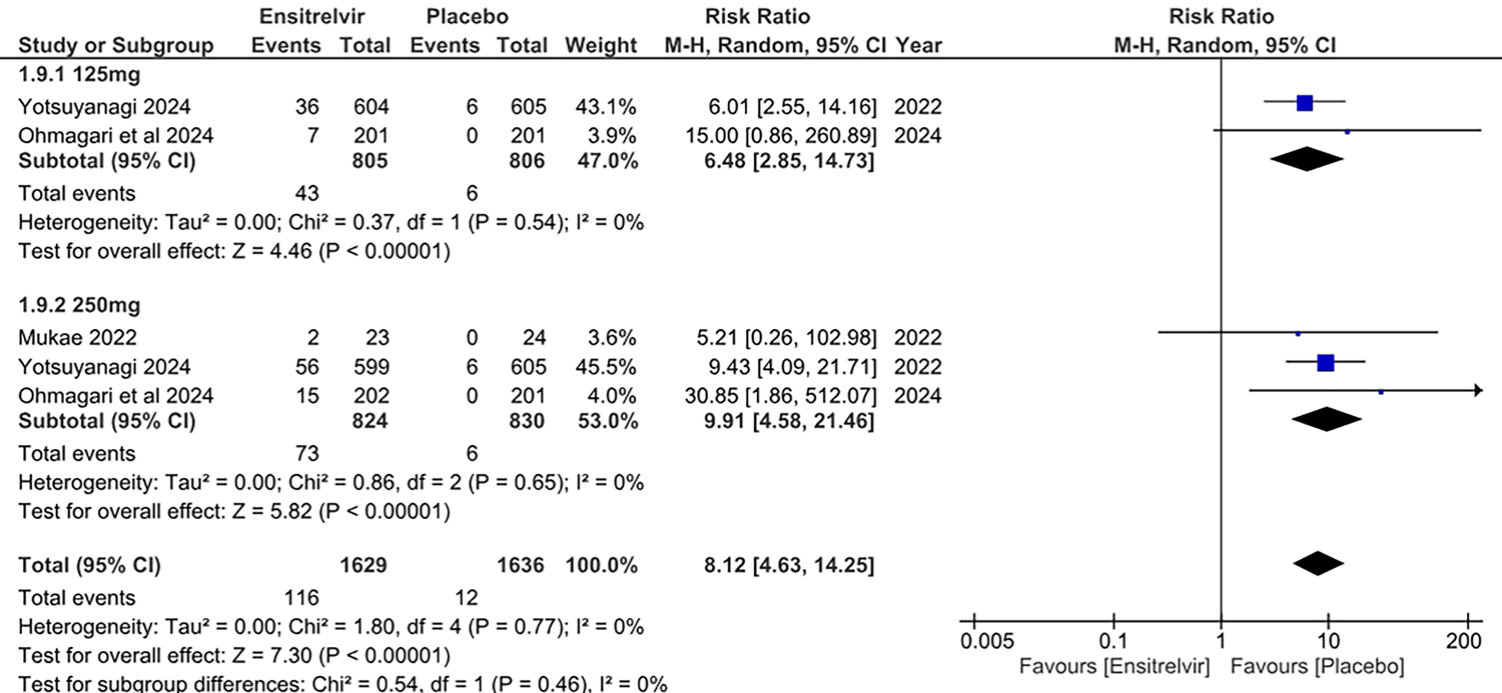
Forest plot of the increased bilirubin levels in the Ensitrelvir and placebo groups

Three studies reported increased bilirubin levels as an outcome [10, 13, 16]. The pooled analysis in Fig. 6 showed significant risk of increase in bilirubin levels with Ensitrelvir treatment as compared to placebo, for both 125 mg dose [RR: 6.48; 95% CI 2.85 to 14.73; p < 0.01; I^2^ = 0%] and 250 mg dose [RR: 9.91; 95% CI 4.58 to 21.46; p < 0.01; I^2^ = 0%]. The combined analysis revealed similar findings [RR: 8.12; 95% CI 4.63 to 14.25; p < 0.01; I^2^ = 0%]. No heterogeneity was observed among the studies.

#### Increased aspartate aminotransferase (AST) levels

**Fig. 7:**
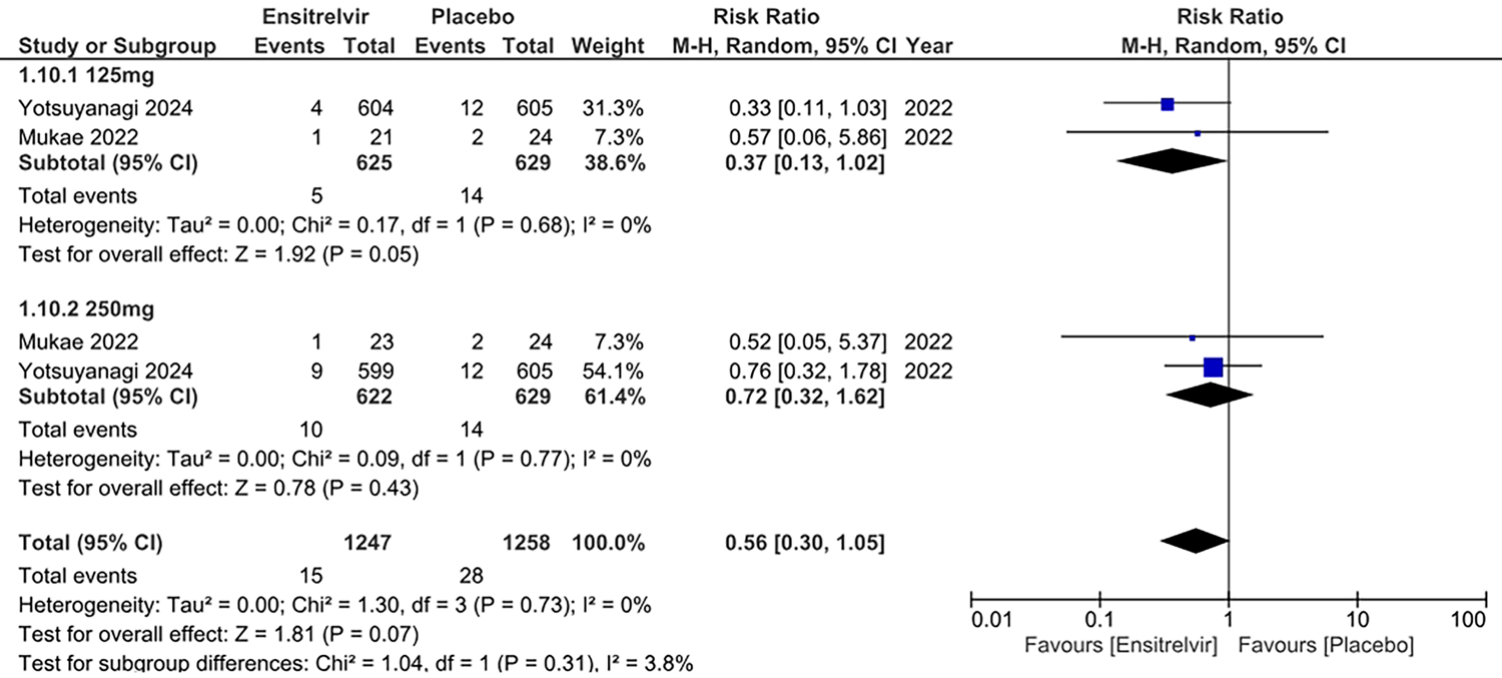
Forest plot of the increased AST levels in the Ensitrelvir and placebo groups

Two studies reported increased AST levels as an outcome [10, 16]. The pooled analysis in Fig. 7 showed a significantly reduced risk of Increased AST for 125 mg Ensitrelvir [RR: 0.37; 95% CI 0.13 to 1.02; p = 0.05; I^2^ = 0%] compared to placebo. But there was no difference between Ensitrelvir 250 mg dose [RR: 0.72; 95% CI 0.32 to 1.62; p = 0.43; I^2^ = 0%] and placebo. The overall analysis indicated no significant difference in the risk of increased AST levels with Ensitrelvir compared to placebo [RR: 0.56; 95% CI 0.30 to 1.05; p = 0.07; I^2^ = 0%]. No heterogeneity was observed among the studies.

### Headache

**Fig. 8:**
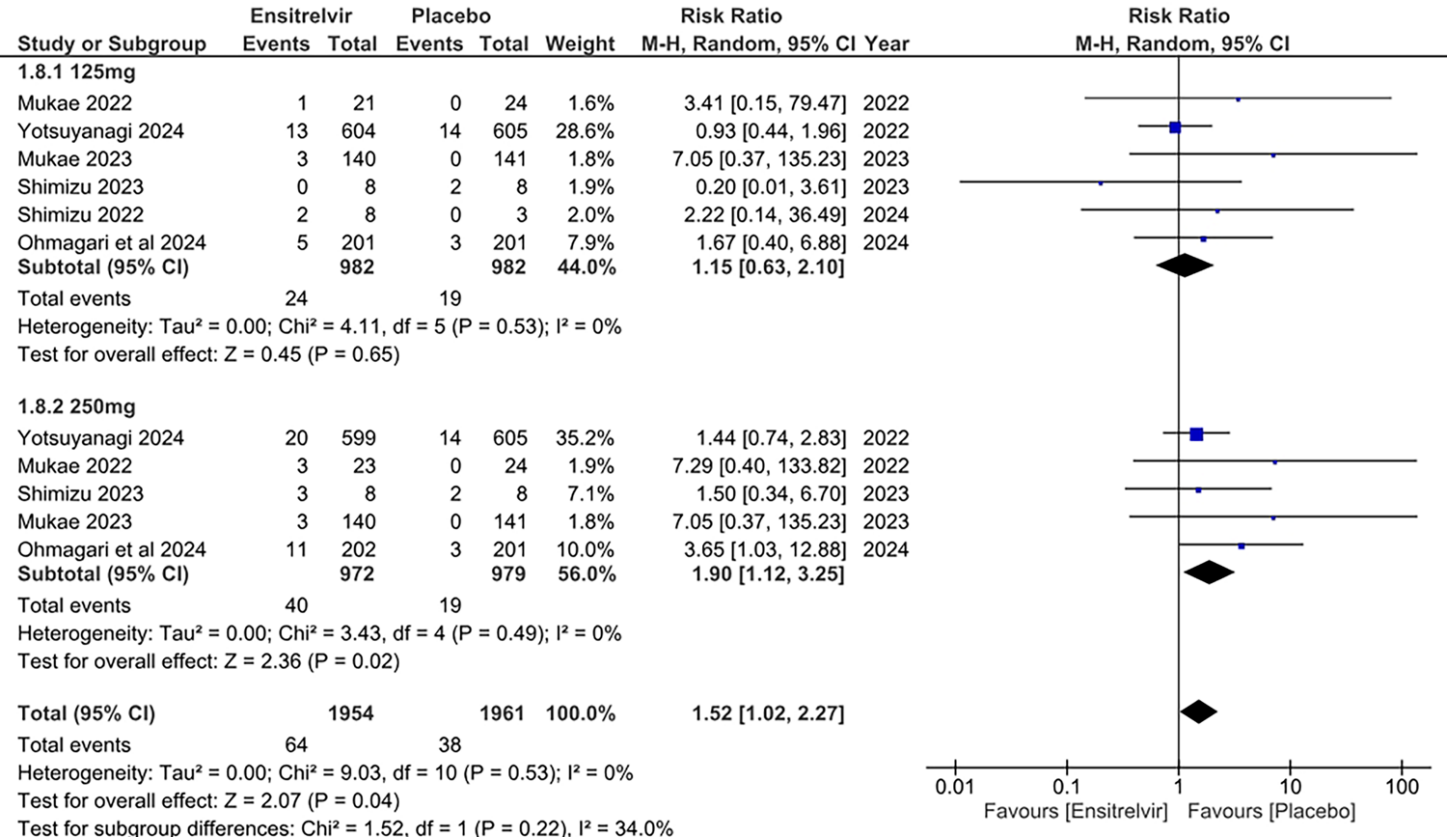
Forest plot of the incidence of headaches in the Ensitrelvir and placebo groups

Six studies investigated the incidence of headaches in patients consuming Ensitrelvir at different doses (125 and 250 mg) compared to placebo [10, 13–17]. The pooled analysis in Fig. 8 demonstrated no significant difference in headache occurrence between the 125 mg dose of Ensitrelvir [RR: 1.15; 95% CI 0.63 to 2.10; p = 0.65; I^2^ = 0%] and placebo. However, the 250 mg dose was associated with a significantly higher incidence compared to placebo [RR: 1.90; 95% CI 1.12 to 3.25; p = 0.02; I^2^ = 0%] The overall analysis found a significant increase in the risk of headache for those taking Ensitrelvir compared to placebo [RR: 1.52; 95% CI 1.02 to 2.27; p = 0.04; I^2^ = 0%]. No heterogeneity was observed among the studies.

### Diarrhea

**Fig. 9:**
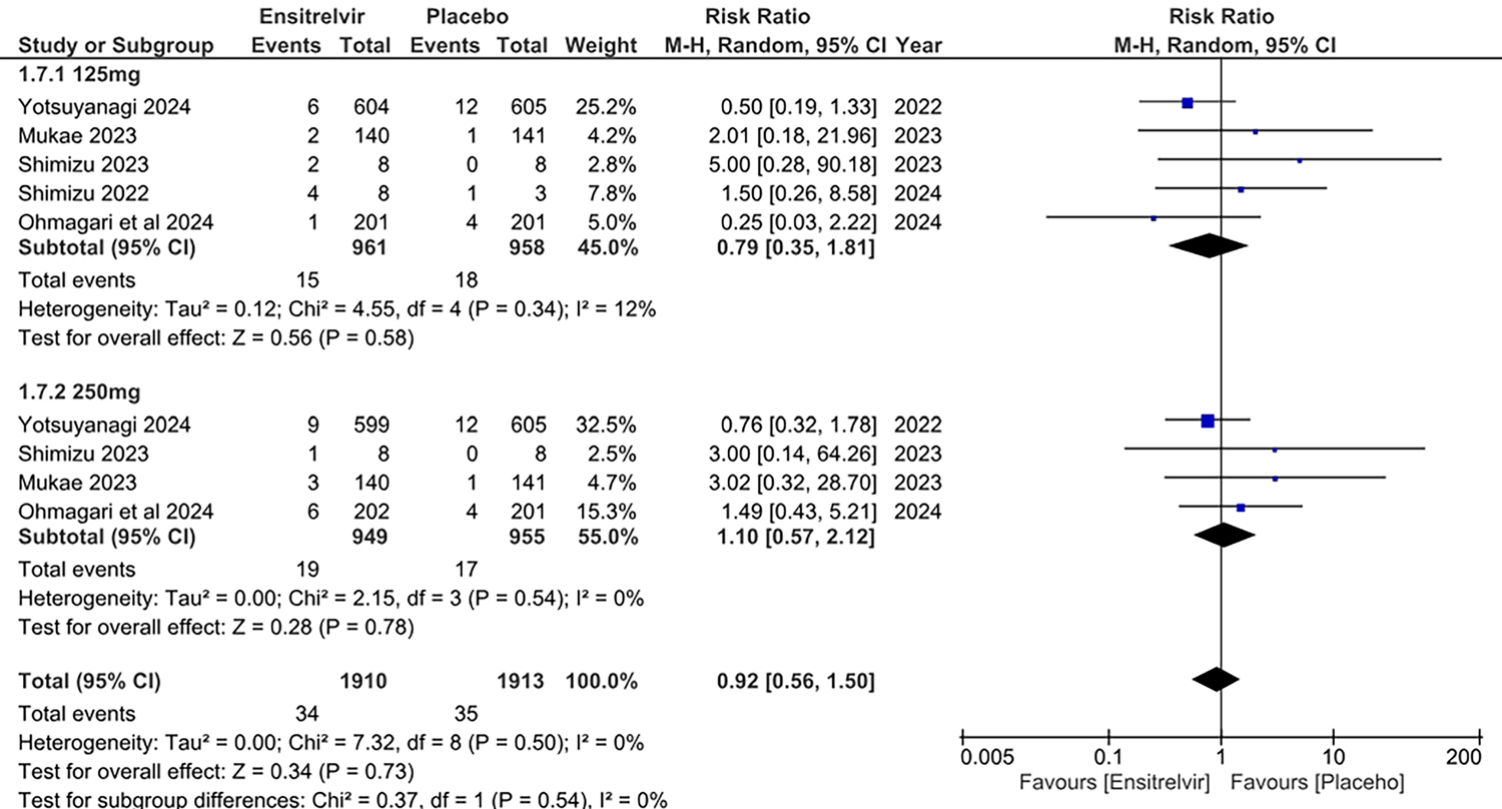
Forest plot of the incidence of diarrhea in the Ensitrelvir and placebo groups

Five studies reported the incidence of diarrhea in Ensitrelvir at different doses (125 and 250 mg) compared to placebo [10, 13–15, 17]. The pooled analysis in the Fig. 9 showed no significant difference in risk of diarrhea between Ensitrelvir and placebo for both 125 mg dose [RR: 0.79; 95% CI 0.35 to 1.81; p = 0.58; I^2^ = 12%] and 250 mg dose [RR: 1.10; 95% CI 0.57 to 2.12; p = 0.54; I^2^ = 0%]. The overall analysis combining both doses followed the same trend [RR: 0.92; 95% CI 0.56 to 1.50; p = 0.73; I^2^ = 0%]. No overall heterogeneity was observed among the studies.

### Treatment-emergent adverse events (TEAEs)

**Fig. 10:**
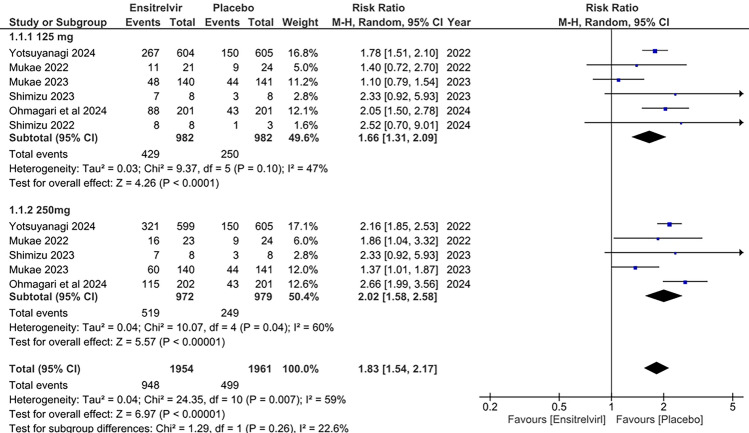
Forest plot of the TEAEs in the Ensitrelvir and placebo groups

Six studies reported the incidence of TEAEs associated with Ensitrelvir at different doses (125 and 250 mg) [10, 13–17]. According to the pooled analysis in Fig. 10, There was high risk of TEAEs with Ensitrelvir for both 125 mg dose [RR: 1.66; 95% CI 1.31 to 2.09; p < 0.01; I^2^ = 47%] and 250 mg dose [RR: 2.02; 95% CI 1.58 to 2.58; p < 0.01; I^2^ = 60%] compared to placebo. The overall effect similarly illustrated a significant increase in the risk of TEAEs with Ensitrelvir compared to placebo [RR: 1.83; 95% CI 1.54 to 2.17; p < 0.01; I^2^ = 59%]. An overall moderate heterogeneity was observed, which was reduced to 0% after leaving out Mukae 2023 (Supplementary Fig. 3) [17]

### Treatment-related adverse events (TRAEs)

**Fig. 11:**
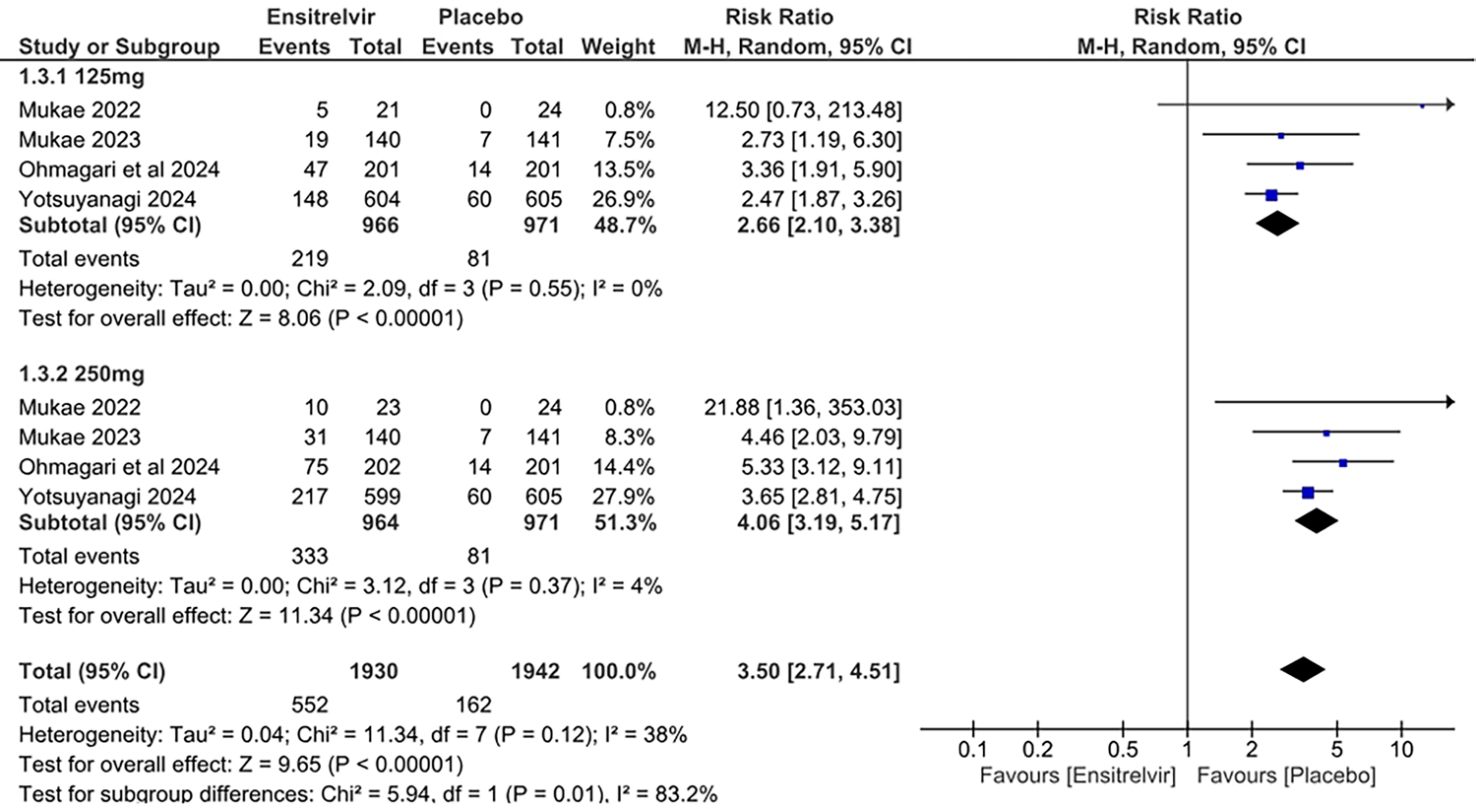
Forest plot of the Treatment-related AEs in the Ensitrelvir and placebo groups

Four studies reported the TRAEs as an outcome [10, 13, 16, 17]. The pooled analysis in Fig. 11 showed significantly higher risk of TRAEs in patients taking Ensitrelvir compared to placebo; both 125 mg dose of Ensitrelvir [RR: 2.66; 95% CI 2.10 to 3.38; p < 0.01; I^2^ = 0%] and 250 mg dose [RR: 4.06; 95% CI 3.19 to 5.17; p < 0.01; I^2^ = 4%] demonstrated this effect. The overall analysis illustrated similar findings with a higher risk of TRAEs in the Ensitrelvir group [RR: 3.50; 95% CI 2.71 to 4.51; p < 0.01; I^2^ = 38%]. Moderate heterogeneity (I^2^ = 38%) was observed overall across the studies, which was reduced to zero after leaving out Yotsuyanagi [10]

### Serious treatment-emergent adverse events (TEAE)

**Fig. 12:**
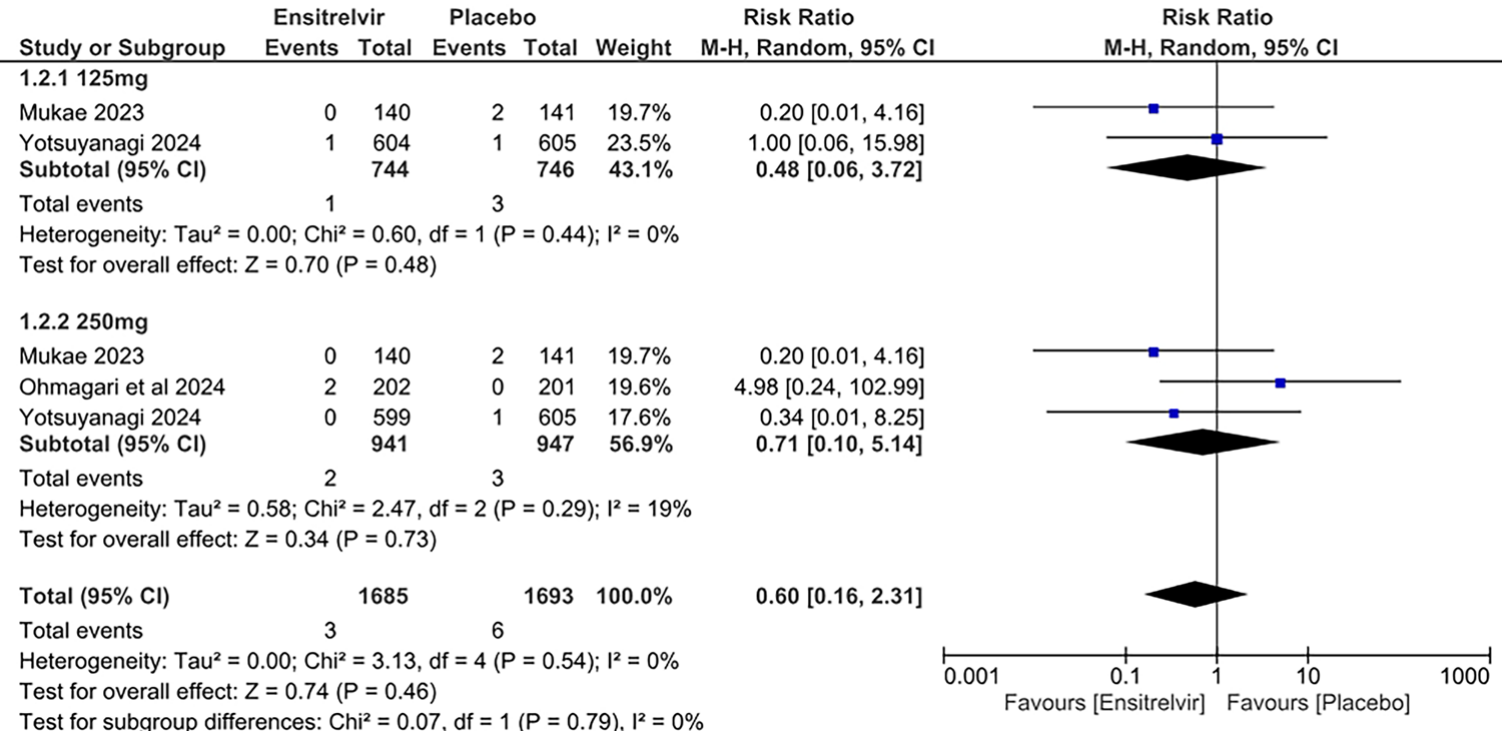
Forest plot of the serious TEAEs in the Ensitrelvir and placebo groups

Three studies reported serious TEAEs as an outcome [10, 13, 17]. The pooled analysis in Fig. 12 demonstrated no significant difference in the occurrence of serious TEAEs between patients taking Ensitrelvir and placebo. Both the 125 mg dose [RR: 0.48; 95% CI 0.06 to 3.72; p = 0.48; I^2^ = 0%] and the 250 mg dose [RR: 0.71; 95% CI 0.10 to 5.14; p = 0.73; I^2^ = 19%] of Ensitrelvir supported this effect. The overall analysis similarly indicated no significant difference in the risk of serious TEAEs with Ensitrelvir compared to placebo [RR: 0.60; 95% CI 0.16 to 2.31; p = 0.46; I^2^ = 0%]. No overall heterogeneity was observed among the studies.

### Adverse events (AEs) leading to treatment discontinuation

**Fig. 13:**
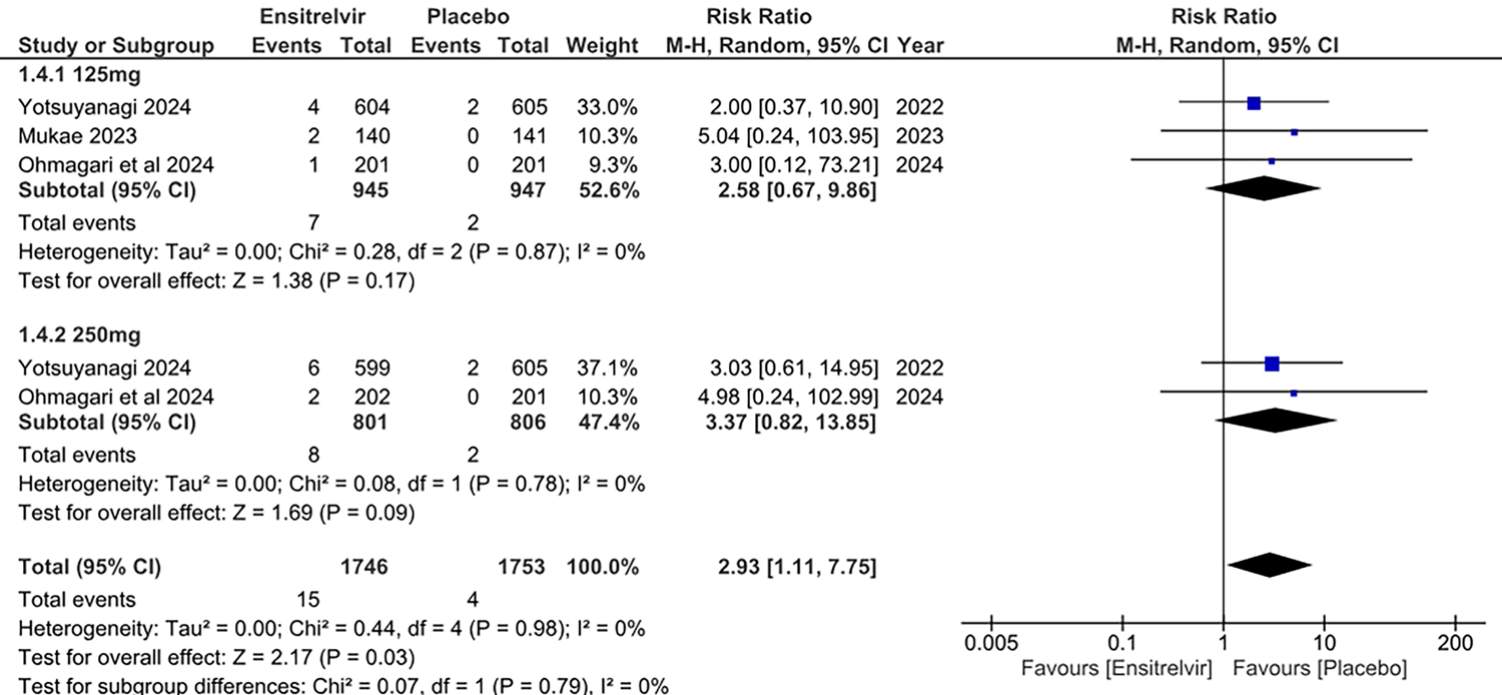
Forest plot of the AEs leading to treatment discontinuation in the Ensitrelvir and placebo groups

Three studies reported AEs that led to treatment discontinuation as an outcome [10, 13, 17]. The combined analysis in Fig. 13 revealed no significant difference between the Ensitrelvir and placebo groups regarding AEs leading to treatment discontinuation for both the 125 mg dose [RR: 2.58; 95% CI 0.67 to 9.86; p = 0.17; I^2^ = 0%] and the 250 mg dose [RR: 4.37; 95% CI 0.82 to 13.85; p = 0.09; I^2^ = 0%]. However, the overall analysis indicated a significantly higher risk of AEs leading to treatment discontinuation with Ensitrelvir compared to placebo [RR: 2.93; 95% CI 1.11 to 7.75; p = 0.03; I^2^ = 0%]. No heterogeneity was observed among the studies.

## Discussion

To the best of our knowledge, this is the first meta-analysis to evaluate the efficacy and safety of Ensitrelvir in patients with COVID-19. Our findings demonstrate that Ensitrelvir significantly reduces SARS-CoV-2 viral RNA levels compared to placebo, suggesting its potential antiviral efficacy. However, the drug was associated with a higher risk of decreased blood HDL levels, increased triglyceride and bilirubin levels, and treatment-related adverse events (TRAEs). No significant differences were observed in the incidence of diarrhea, serious treatment-emergent adverse events (TEAEs), or adverse events leading to treatment discontinuation. However, there was a higher overall risk of TEAEs and headaches, especially at the 250 mg dose. The findings generally show consistent results.

The significant reduction in SARS-CoV-2 viral RNA levels with Ensitrelvir aligns with the proposed antiviral mechanism of the drug, which acts as a 3-chymotrypsin-like protease inhibitor, the main protease found in coronaviruses. The consistent effect across studies suggests a robust antiviral activity [16, 18]. Notably, the pooled analysis for viral RNA levels showed moderate heterogeneity (I^2^ = 28%), which was reduced to zero after excluding Mukae 2022, indicating that this study significantly influenced the favorable efficacy outcome [16]. Mukae 2022 reported a larger effect size for viral RNA reduction, potentially due to factors such as its study population (e.g., predominantly Asian patients with mild-to-moderate COVID-19), dosing regimen (e.g., 125 mg or 250 mg Ensitrelvir), or timing during the Omicron variant wave [16]. This antiviral activity is comparable to that of nirmatrelvir/ritonavir (Paxlovid), an approved oral antiviral for mild-to-moderate COVID-19, which also inhibits the SARS-CoV-2 main protease [19]. However, Ensitrelvir offers potential advantages over Paxlovid. As a single-agent therapy, Ensitrelvir eliminates the need for ritonavir, which is associated with significant drug-drug interactions, particularly in patients with comorbidities or those on multiple medications [20]. This simpler administration profile may improve patient adherence and broaden applicability, especially in outpatient settings.

Similarly, a study conducted on the efficacy and safety of Ensitrelvir in treating COVID-19 from February 10 to July 10, 2022, with a 28-day follow-up period, at 92 institutions in Japan, Vietnam, and South Korea has shown promising results where it demonstrated significant efficacy by reducing the median time to symptom resolution by approximately one day compared to placebo. [10]

However, it is essential to consider the limitations of viral load reduction as a sole surrogate marker for clinical outcomes. Previous clinical trials of oral antivirals for COVID-19, molnupiravir and nirmatrelvir, conducted during the Delta variant phase in unvaccinated high-risk adults, showed a reduction in hospitalization and death [21, 22]. However, with COVID-19 vaccinations and the emergence of the Omicron variant, these endpoints are less applicable since studies have indicated a lower risk of severe outcomes with Omicron compared to Delta, [23], and community-based research suggests limited antiviral effectiveness in vaccinated, high-risk adults or those infected with Omicron [24]. It must be noted that studies conducted on symptomatic relief as an endpoint are guided by the FDA's 2020 recommendation to implement patient-reported outcomes for measuring COVID-19 symptoms in drug trials [25]. The phase 2/3 EPIC-SR study tested ritonavir-boosted nirmatrelvir for symptom alleviation but found no significant difference [26]. However, the SCORPIO-SR study demonstrated Ensitrelvir 's effectiveness in resolving COVID-19 symptoms, regardless of risk factors. Ensitrelvir showed symptom relief and virus reduction benefits for mild to moderate cases. Further trials, including SCORPIO-HR and STRIVE, [27, 28] and a pediatric study, are underway to validate its efficacy across different patient groups. While both nirmatrelvir and Ensitrelvir aim to alleviate COVID-19 symptoms, differing outcomes in symptom relief may stem from differences in trial design, patient populations, timing of administration, and pharmacokinetics. For instance, Ensitrelvir's single-agent nature and distinct protease-binding profile might contribute to the more rapid symptom resolution observed in specific trials.

The findings of this meta-analysis suggest that Ensitrelvir has antiviral activity against SARS-CoV-2, where RNA load has decreased. While Ensitrelvir significantly reduces SARS-CoV-2 viral RNA levels, its clinical significance remains uncertain, particularly for lowering hospitalization or mortality. Viral load reduction may not always translate to clinical benefits, as seen with molnupiravir and nirmatrelvir in vaccinated or Omicron-infected populations. [21, 22, 24, 26]. Moreover, the significant safety concerns outweigh the potential benefits based on the current evidence. The drug was associated with a significantly increased risk of several adverse events, including lipid abnormalities and liver enzyme elevations. These findings highlight the potential for metabolic disturbances and hepatotoxicity with Ensitrelvir use. While the incidence of serious adverse events was not significantly increased, the overall adverse event profile is unfavorable. Contrastingly, Yotsuyanagi et al. demonstrate that no treatment-related serious adverse events were reported [10].While Ensitrelvir’s single-agent formulation and preliminary efficacy in vaccinated or Omicron-infected patients are promising, its unfavorable adverse event profile limits claims of superiority over Paxlovid.

Our meta-analysis has multiple limitations. Firstly, only 6 studies were included, which limits the accuracy of our findings, and they were only RCTs, excluding potentially valuable insights from non-randomized studies, including observational studies and case reports, which could offer broader context or reveal findings not captured by RCTs alone. Furthermore, the predominance of Asian patients limits the generalizability of our findings to other racial and ethnic groups, as differences in genetics, comorbidities, or healthcare settings may influence Ensitrelvir’s efficacy and safety profiles. Incorporating data from a broader range of study designs could offer more comprehensive insights. Furthermore, despite efforts to mitigate publication bias, relying on published literature introduces an element of publication bias, which indicates the need to conduct further trials on the drug rather than solely relying on the published literature to evaluate the efficacy and safety of the drug. Expanding the search to include unpublished data from trial registries could also improve the robustness of the findings. Additionally, the variability in how efficacy and safety endpoints are reported potentially contributes to heterogeneity between studies, which could be mitigated by establishing standardized reporting guidelines for these outcomes across studies. Our meta-analysis also focused on specific adverse events and the limited literature published. Thus, it may not have captured the full spectrum of potential side effects, highlighting the importance of further research. Lastly, relying on the Cochrane RoB tool may be open to subjective interpretation, which could be reduced by having multiple independent reviewers assess each study and reach a consensus on any disagreements.

The role of Ensitrelvir in the treatment algorithm remains uncertain due to the limited available data and the significant safety concerns. However, our findings are encouraging and warrant further research. Further research is needed to identify patient subgroups who may benefit from Ensitrelvir and to develop strategies to mitigate the adverse effects. Until more data becomes available, Ensitrelvir should be approached with caution and careful risk–benefit assessment. Research should focus on evaluating the impact of Ensitrelvir on clinical endpoints such as hospitalization and mortality. The long-term adverse effects potentially associated with this drug must also be further explored to highlight its safety profile more comprehensively.

## Conclusion

This meta-analysis and systematic review provide evidence for the antiviral efficacy of Ensitrelvir in reducing SARS-CoV-2 viral load. Still, its impact on hospitalization and mortality is unclear due to limited data. However, the drug's unfavorable safety profile, characterized by a significant increase in adverse events, limits its clinical utility. Further research is warranted to explore the potential benefits and risks of Ensitrelvir in specific patient populations.

## Supplementary Information

Below is the link to the electronic supplementary material.

Supplementary file1 (DOCX 309 KB)

## Data Availability

No datasets were generated or analysed during the current study.

## Abbreviations

SARS-CoV-2: Severe acute respiratory syndrome coronavirus 2
ARDS: Acute respiratory distress syndrome
RCT: Randomized controlled trial
AEs: Adverse events
HDL: High-density lipoproteins
AST: Aspartate aminotransferase
TEAEs: Treatment-emergent adverse events
TRAEs: Treatment-related adverse events
