# Supplementary material for: Efficacy and safety of Ensitrelvir in asymptomatic or mild to moderate COVID-19: a systematic review and meta-analysis of randomized controlled trials
Source: Infection. 2025 Jul 31;53(5):1645–61. doi: 10.1007/s15010-025-02582-0 (PMC12460428; doi:10.1007/s15010-025-02582-0)
Supplement: Supplementary file 1 — Supplementary file1 (DOCX 309 KB) [file 15010_2025_2582_MOESM1_ESM.docx]

**Table S1: Detailed Search Strategy**

| **DATABASE** | **SEARCH STRATEGY** | **SEARCH RESULTS** |
| --- | --- | --- |
| **Pubmed** | ((("ensitrelvir"[Supplementary Concept] OR "ensitrelvir"[All Fields] OR ("ensitrelvir"[Supplementary Concept] OR "ensitrelvir"[All Fields] OR "s 217622"[All Fields]) OR "px665raa3h"[EC/RN Number] OR ("ensitrelvir"[Supplementary Concept] OR "ensitrelvir"[All Fields])) AND ("ensitrelvir"[Supplementary Concept] OR "ensitrelvir"[All Fields] OR "s 217622"[All Fields])) OR ("3CL"[All Fields] AND ("protease inhibitors"[Pharmacological Action] OR "protease inhibitors"[MeSH Terms] OR ("protease"[All Fields] AND "inhibitors"[All Fields]) OR "protease inhibitors"[All Fields] OR ("protease"[All Fields] AND "inhibitor"[All Fields]) OR "protease inhibitor"[All Fields]))) AND ("COVID-19"[MeSH Terms] OR "SARS-CoV-2"[MeSH Terms] OR "Post-Acute COVID-19 Syndrome"[MeSH Terms] OR "COVID-19"[All Fields] OR "SARS-CoV-2"[All Fields] OR "coronavirus disease 2019"[All Fields] OR ("Post-Acute COVID-19 Syndrome"[MeSH Terms] OR ("post acute"[All Fields] AND "COVID-19"[All Fields] AND "syndrome"[All Fields]) OR "Post-Acute COVID-19 Syndrome"[All Fields] OR "post covid 19 condition"[All Fields])) | 577 |
| **Google scholar** | (Ensitrelvir) OR (s 217622) OR (px665raa3h) AND (COVID-19) OR (SARS-CoV-2) OR (coronavirus disease 2019) OR (Post-Acute COVID-19 Syndrome") | 357 |
| **Cochrane** | **#1:** (Ensitrelvir):ti,ab,kw OR (s 217622):ti,ab,kw  **#2:** (COVID-19):ti,ab,kw OR (SARS-CoV-2):ti,ab,kw OR (coronavirus disease 2019):ti,ab,kw OR (Post-Acute COVID-19 Syndrome):ti,ab,kw  **#1 AND #2** | 28  21381  28 |

**Figure S1**: The risk of bias table for the included trials.


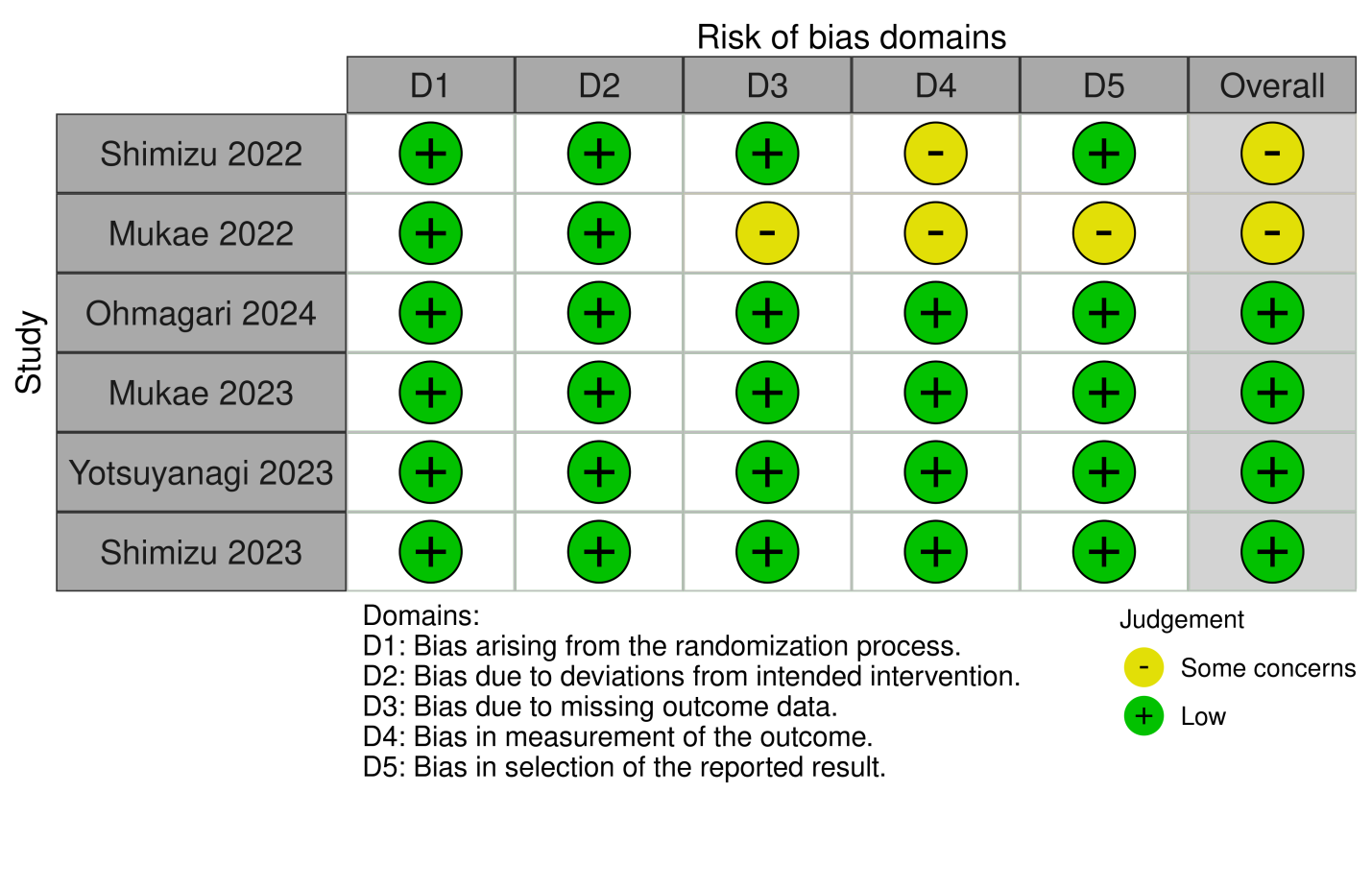


**Figure S2**: The risk of bias summary for the included trials.


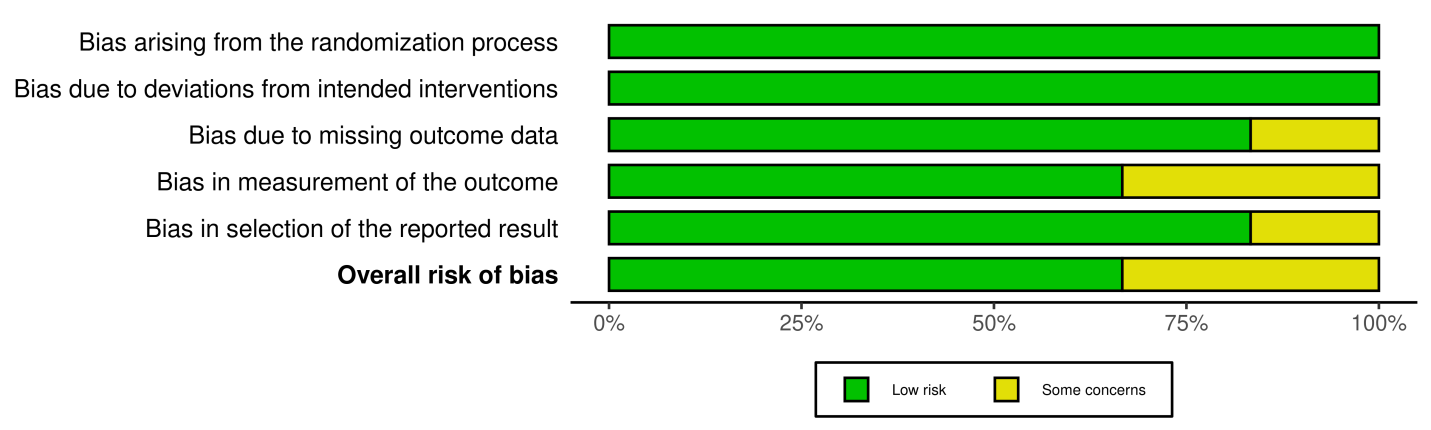


**Figure S3:** The traffic light plot for the included trials.


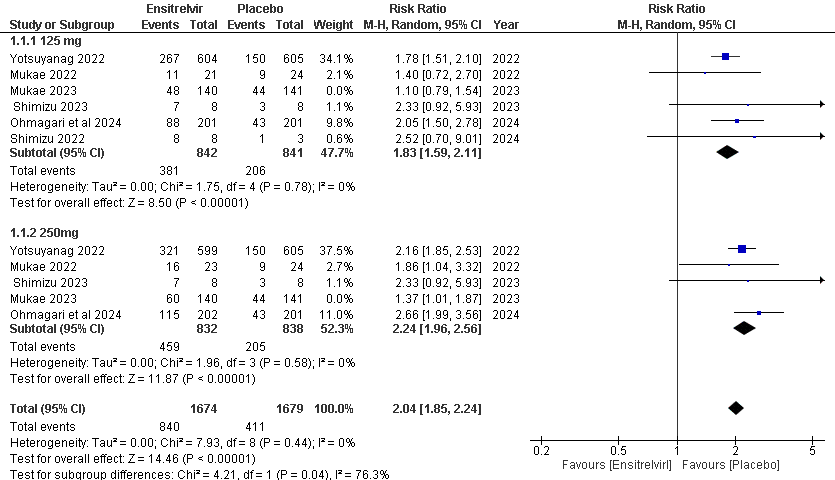


**Figure S3:** Forest plot of the TEAEs in the Ensitrelvir and Placebo groups after performing sensitivity analysis excluding Mukae et al 2023

**Table S2**: GRADE Evidence Profile, Ensitrelvir compared to Placebo for Mild to Moderate COVID-19 Infection

| **Outcome** | **№ of studies** | **№ of pa- tients** | **Relative effect (95% CI)** | **Absolute**  **(95% CI)** | **Certainty** | **Comments** |
| --- | --- | --- | --- | --- | --- | --- |
| SARS-CoV-2 | 3 | 1535 | MD: -1.35 | - | ✦✦✦ | Downgraded one level |
| Viral RNA Levels |  |  | (-1.58 to |  | Moderate | for inconsistency due |
|  |  |  | -1.13) |  |  | moderate heterogeneit |
|  |  |  |  |  |  | (I² = 28%) which was |
|  |  |  |  |  |  | resolved after |

# effect

Decrease in Blood Cholesterol Levels

Decrease in Blood HDL Levels

## 2 2412 RR: 8.83

(4.05 to

19.27)

## 6 2932 RR: 9.25

(7.25 to

11.81)

## - ✦✦✦

Moderate

- ✦✦✦✦

High

sensitivity analysis. Downgraded one level for imprecision due to wide confidence interval (4.05 to 19.27 Low heterogeneity (I²

= 1%), no serious concerns for risk of bias, indirectness, or imprecision.

Increase in Blood

## 5 2896 RR: 2.00

- ✦✦✦✦

No heterogeneity (I² =

Triglyceride Levels

Increase in Bilirubin Levels

Increase in AST Levels

(1.55 to

2.57)

## 3 2459 RR: 8.12

(4.63 to

14.25)

## 2 1876 RR: 0.56

(0.30 to

High

## - ✦✦✦

Moderate

## - ✦✦

Low

0%), no serious concerns for risk of bias, indirectness, or imprecision.

Downgraded one level for imprecision due to wide confidence interval (4.63 to 14.25 Downgraded one level for imprecision due to

1.05) wide confidence interv

(0.30 to 1.05) that includes the null effect (RR = 1), and one level for small number of studies (n=2).

Headache 6 2936 RR: 1.52

(1.02 to

2.27)

Diarrhea 5 2868 RR: 0.92

(0.56 to

1.50)

## - ✦✦✦

Moderate

## - ✦✦✦

Moderate

Downgraded one level for imprecision due to wide confidence interval (1.02 to 2.27) that includes the null effect (RR = 1).

Downgraded one level for imprecision due to wide confidence interval (0.56 to 1.50) that includes the null effect (RR = 1).

# effect

| **Outcome** | **№ of studies** | **№ of pa- tients** | **Relative effect (95% CI)** | **Absolute**  **(95% CI)** | **Certainty** | **Comments** |
| --- | --- | --- | --- | --- | --- | --- |
| Treatment- | 6 | 2936 | RR: 1.83 | - | ✦✦✦ | Downgraded one level |
| Emergent |  |  | (1.54 to |  | Moderate | for inconsistency due |
| Adverse Events |  |  | 2.17) |  |  | moderate heterogeneit |
| (TEAEs) |  |  |  |  |  | (I² = 59%) which was |
|  |  |  |  |  |  | resolved after |

Treatment- Related Adverse

## 4 2901 RR: 3.50

(2.71 to

## - ✦✦✦

Moderate

sensitivity analysis. Downgraded one level for inconsistency due

Events (TRAEs)

4.51)

moderate heterogeneit

Serious TEAEs 3 2632 RR: 0.60

(0.16 to

## - ✦✦

Low

(I² = 38%) which was resolved after sensitivity analysis.

Downgraded one level for imprecision due to

2.31) wide confidence interv

(0.16 to 2.31) that includes the null effect (RR = 1), and one level for small number of studies (n=3).

AEs Leading to Treatment Discontinuation

## 3 2693 RR: 2.93

(1.11 to

7.75)

## - ✦✦✦

Moderate

Downgraded one level for imprecision due to wide confidence

interval (1.11 to 7.75).

*Note:* Certainty ratings: ✦✦✦✦ = High, ✦✦✦ = Moderate, ✦✦ = Low, ✦ = Very Low.
